# Unveiling why race does not affect the mask effect on attractiveness: but gender and expression do

**DOI:** 10.1186/s41235-024-00534-0

**Published:** 2024-02-14

**Authors:** Ellie Hewer, Michael B. Lewis

**Affiliations:** School of Psychology, Cardiff University, Park Place, Cardiff, UK

**Keywords:** Mask effects, Attractiveness, Race, Gender, Face expressions

## Abstract

Studies show that surgical face masks can have both positive and negative effects on attractiveness. Race has been implicated as a moderator of the size of this mask effect. Here, the moderating effects of expression, race and gender are explored. The mask effect was more positive for males than for females, for neutral faces than for smiling faces, and there were differences between the races. Further, the effect of unmasked attractiveness was partialled out for each image, which removed the race effects, but the gender and expression effects remained. It is suggested that racial differences previously observed in the mask effects are a consequence of differences in attractiveness of the faces sampled from those races. Re-analysis of previous research that showed race effects also demonstrates how they are better explained as attractiveness effects rather than race effects. This explanation can provide order to the different findings observed across the literature.

## Introduction

The wearing of a face mask has many effects on the processing of faces and of particular interest here is the effect that it has on the perceived attractiveness of the face. Miyazaki and Kawahara (2016) first investigated the effects of masks on attractiveness and showed that in a sample of Japanese women, masking made faces appear less attractive. The argument put forward to explain Miyazaki and Kawahara’s (2016) finding was that the masks were seen as being associated with disease and so the person was seen as a potential disease vector.

The covid pandemic greatly increased the wearing of medical masks in public worldwide and also the research conducted on attitudes towards the wearing of masks. Research has shown that attitudes towards masks prior to the pandemic were that mask wearers were unhealthy; however, participants taking part in the Kamatani et al. (2021) study reported they felt neutral towards mask wearers or thought they were healthy. Further research suggests the social perceptions of mask wearers were more positive than those who were not wearing masks (Olivera-La Rosa et al., 2020). It has also been shown that positive views on masks are linked to increased mask wearing and increased attractiveness perceptions (Dudarev et al., 2021), which has implications for future mask interventions and adherence. This suggests that the Coronavirus pandemic led to new norms about masks, and mask wearers were viewed as more socially acceptable as they were upholding these new norms to protect others.

Research carried out during and after the covid pandemic reveals a different effect of face masks on attractiveness than first found by Miyazaki and Kawahara (2016). Hies and Lewis (2022) conducted their study during the height of the pandemic and found that in White male faces, masks increased the perceived facial attractiveness. This study also showed that this effect was strongest for medical masks compared to other occluding stimuli. Similarly, Patel et al. (2020) supports these findings and found that in a heterogeneous sample of male and female faces, masks had the biggest improvement on the unattractive faces. In this study, all the faces in the unattractive group were rated as significantly more attractive when masked. The researchers suggested there was an occlusion effect which caused these findings, as the mask tends to hide unattractive features which make the faces appear more attractive. It has also been suggested that masks decrease the attractiveness for more attractive faces while increasing the attractiveness of less attractive faces (Bassiri-Tehrani et al., 2022). This study also found an attractiveness effect for the most attractive and unattractive female faces where the effect was stronger than the male counterparts. This could be argued to be due to masks making faces appear more average, and therefore for unattractive faces, this makes them more attractive, while the opposite is seen for attractive faces. This highlights that there may also be important gender factors which interact with the mask effect. These studies collectively suggest a pattern in which less attractive faces are made more attractive with a mask, whereas more attractive faces are made less attractive with a mask—although that pattern was not observed by Hies and Lewis (2022).

### Masks and the effect of race

Race or ethnicity have been argued to affect the perception of medical masks. For example, Fearnley and Wu (2022) found that there were differences between Western and Asian communities regarding their attitudes to masks. This could potentially explain the differences that have been shown in the mask effect on attractiveness of faces when looked across races. Dudarev et al. (2022), for example, found that adding a mask to White faces made them appear more attractive; however, adding a mask to Asian faces made them less attractive. This appears to support the contrasting findings from Miyazaki and Kawahara (2016) and Hies and Lewis (2022), suggesting that there is an interaction between race and masks, which could be causing the different findings.

Kamatani et al. (2023) explored this race effect on the mask attractiveness advantage and found that masks improve facial attractiveness for Black faces and White faces but not for Japanese faces. Their explanation was based on the fact that Black and White faces were other-race for the participants whereas Japanese faces were same-race. The same-race faces are perceived as less attractive when masked due to high exposure to those faces, compared to other-race faces where there is less exposure to unattractive faces of that race and over exposure to highly attractive faces such as celebrities. This means that when imagining what is behind the mask, the imagined other-race face will be more attractive than the imagined same-race face.

An alternative explanation for the race effects on the mask advantage is presented here, based on the base attractiveness levels of the groups of faces used in the experiments. It is hypothesised that more attractive faces will show less of a mask advantage and, further, the faces selected to represent the different racial groups may differ in their base attractiveness. The differences in attractiveness between the racial groups may be a matter of unmatched selection or it could be a systematic difference in general attractiveness between races. The latter possibility is supported by observations that there are differences in average attractiveness for different races (e.g. Lewis, 2011, 2012), and these differences are gender-based such that female Asian faces on average tend to be rated as more attractive than female Black faces, whereas male Black faces tend to be rated on average as more attractive than male Asian faces. It is possible that these trends only occur for the majority White raters (as tested in those experiments), but it has been demonstrated that there is good cross-cultural agreement on what makes an attractive face (e.g. Coetzee et al., 2014). Regardless of whether there are universally accepted racial differences in attractiveness, it is possible that the attractive of the items used within the research described affect the relative size of the attractiveness advantage seen with medical masks and any observed effect of race is merely an effect of base attractiveness. This possibility is explored here.

### Masks and emotional expression on perceived attractiveness

Studies have also looked at the effect that masks have on emotion recognition and facial attractiveness of different emotional expressions. Masks have been found to impair emotion recognition, particularly of happiness, but enhance perceptions of attractiveness (Parada-Fernández et al., 2022). This supports the pattern found by many face mask studies that masks do improve attractiveness ratings. In contrast, Hopfensitz and Mantilla (2022) found that smiles behind face masks are detectable by others, but similarly improve ratings of attractiveness and trustworthiness in individuals. However, they found this is the case in individuals with 18-month experience with face masks. This highlights the importance of experience which may have an impact on research post-pandemic as people have had greater experience with face masks than they would have pre-pandemic. In addition, it also demonstrates that smiling faces behind masks can improve ratings of attractiveness despite the occlusion of the smile itself. This suggests the subtle differences in the eye area seen when an individual is smiling are important for determining attractiveness. Previous research looking at the mask effect has tended to use neutral expressions. However, smiles enhance attractiveness (Reis et al., 1990), and as smiles can be seen even under a mask, it was explored here how smiling interacted with the mask effect.

### Why do face masks improve attractiveness?

A typical explanation for why masks make faces look more average is through occluding important areas associated with the perception of attractiveness (Bassiri-Tehrani et al., 2022). The observer fills in the missing part of the face, and this is done using a typical schema that is likely to be more attractive than the actual person’s face—unless they are a very attractive person. This could explain both why attractive faces are perceived as more unattractive when masked and why unattractive faces are perceived as more attractive. It is also suggested that there are differences in the perceptions of attractiveness for men and women. The smile is suggested to be the main component in determining attractiveness for females, whereas for males, it is the hair, smile, and eyes (Godinho et al., 2020). This could explain why there have been differences across studies into the face mask effect for males and females (Hies & Lewis, 2022; Miyazaki & Kawahara, 2016).

Furthermore, studies have also found that for attractive faces when the top half of the face is occluded, leaving only the bottom half visible, the perception of attractiveness is significantly decreased (Pazhoohi & Kingstone, 2022). Interestingly, this effect is not found for the unattractive faces. It was proposed that there was a positivity bias present which suggests in the absence of facial information, the face is perceived as more attractive. This also suggests that the eye area is important in determining the attractiveness of faces and could explain why the face mask effect appears to occur strongly for unattractive faces, but less strongly for attractive faces.

### The current study

This study aimed to investigate the face mask effect post-pandemic while looking at three specific moderating factors. The first moderating factor was race of face. Faces selected as being classified as either Black, White and Korean were used to assess the impact of face masks on attractiveness. The second moderating factor was gender. Both male and female faces were employed to explore whether the mask effect is larger for one group or the other. The final moderating factor was expression. Smiling and neutral faces were employed to explore whether smiling still improves attractiveness with a mask on, but also to evaluate the effect of expression on the face mask effect. Interaction between these moderators was also explored, and a secondary analysis explored how the rated attractiveness of the unmasked face affected the size of the mask effects.

## Method

### Participants

Based on Hies and Lewis (2022) measures of attractiveness, the effect size of the comparison between masked and unmasked faces was *d* = 1.27. So, replicating the effect would require just eight participants. As the current study was exploring how this effect is different under different conditions, the number of participants was increased by a factor of 10. In total, 87 students form psychology department of Cardiff University participated (76 were female, 9 were male, 1 described themselves as either other and 1 declined to give a gender; 78 described themselves a White, 2 as Black, 2 as Asian and 1 declined to indicate an ethnicity). The race and gender of the participants was too uniform to allow for analysis between participant groups and so all mention of race or gender effects refers to the stimuli rather than the participants. Recruitment was via an experiment management system and participants received course credit for taking part. The research was approved by Cardiff University School of Psychology Research Ethics Committee.

### Material

**Fig. 1:**
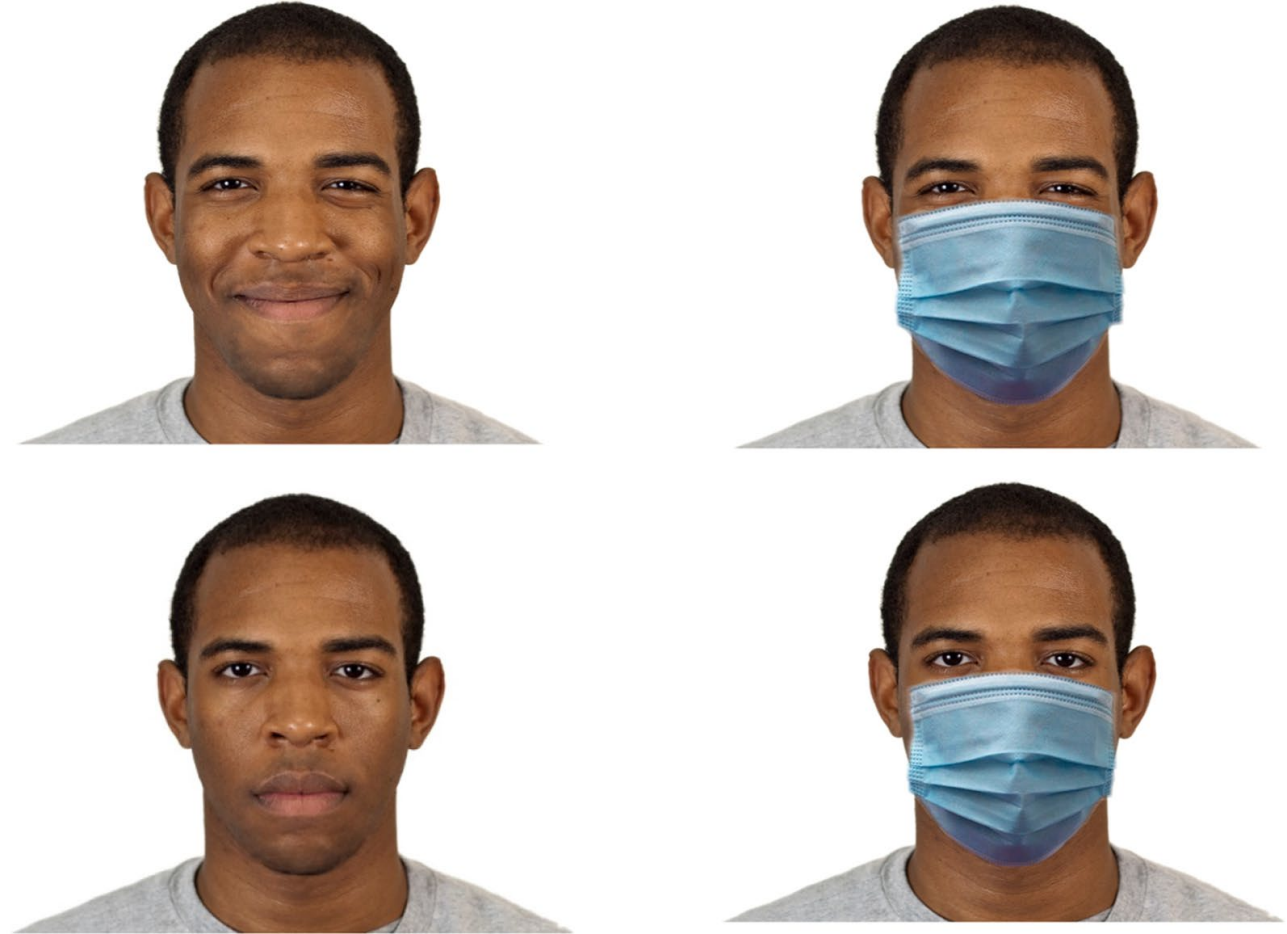
Examples of the stimuli used in the experiment. The left images show the original images smiling (top image) and neutral (bottom image). The right images show the same faces with the medical mask obscuring the lower portion. During the experiment, images were presented individually and in a random order. The original images are reproduced with permission from the Chicago Face Database (Ma et al., 2015) with a Creative Commons Attribution 4.0 (CC BY 4.0) license

Stimuli were obtained from two sources. Black and White faces were taken from the Chicago Face Database (Ma et al., 2015) and Korean faces from the Yonsei Face Database (Chung et al., 2019).^1^ Eight individuals were selected from form each gender for each of the three racial groups. Both the neutral and the closed mouth smile images of the same individuals were used. These images were digitally edited in Corel PHOTO-PAINT to occlude the face with a blue medical mask arranged on the face in a natural manner to look as if it were being worn. Examples of the stimuli are shown in Fig. 1.

### Procedure

The experiment was carried out online using Psychopy 3.0 (Peirce et al., 2019). Participants provided information about their gender and ethnicity and then were presented with a series of 192 images in a random order. For each face, they indicated its attractiveness on a scale from 1 to 7 with higher numbers being more attractive. Participants indicated their ratings by pressing keys 1–7. The experiment was self-paced with an interval 0.25s between the presentation of each face.

### Design

The dependent variable was the attractiveness rating for each image on a scale of 1–7. The independent variables were presence of a mask (masked or not masked) race of the face (Black, White or Korean), gender of the face (male or female) and facial expression (neutral or mouth-closed smile). Order of presentation was fully intermixed and randomised between participants. Participant gender and ethnicity were also recorded as potential covariates although not used in the reported analyses.

## Results and discussion

**Fig. 2:**
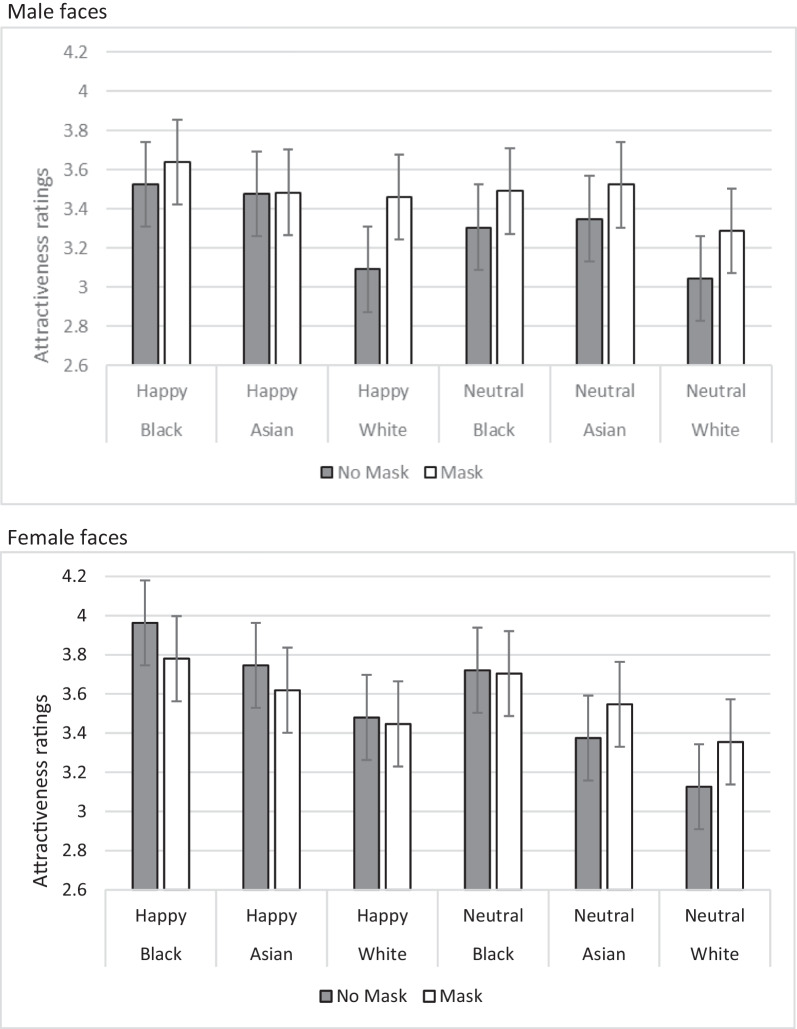
Mean attractive ratings for faces according to race, expression, gender and presence of a mask. Male faces are shown in the top panel and female face in the bottom panel. Error bars show standard errors from the LMM analysis

The 192 ratings from the 87 participants were subjected to a linear mixed models (LMM) analysis. This analysis is superior to ANOVA because it allows both the participants and the faces to be random effects. This provides a more thorough exploration of the entire dataset, rather than an analysis of the variance of generated means. The fixed effects were the properties of the images being: masks, gender, expression, race and interactions between these. Data were analysed using JASP 0.17.1 (JASP team, 2023). The summary of the data is presented in Fig. 2, but as this shows the data split according to all four independent variables, it does not clearly indicate significant findings over groups of items. The AIC of the model was 53,832. The full analysis output is available at https://osf.io/qywxt/.

Looking at the four main effects, masked faces were significantly more attractive than unmasked faces, *F*(1, 16,552) = 26.373, *p* < 0.001, and smiling faces were more attractive than neutral faces, *F*(1, 16,552) = 73.034, *p* < 0.001. Race and gender did not significantly affect attractiveness (note that these were between-face variables, whereas masks and expression were within-item variables).

Of most interest here were the factors that interacted with how masks affect attractiveness. There was a significant interaction of race-by-mask on attractiveness, *F*(2, 16,552) = 8.840, *p* < 0.001: the advantage for wearing a mask was largest for White faces and smallest for Black faces. There was a significant gender-by-mask interaction on attractiveness, *F*(1, 16,552) = 22.624, *p* < 0.001: the advantage for mask wearing was larger for male faces than for female faces. There was a significant interaction of expression-by-mask on attractiveness, *F*(1, 16,552) = 22.624, *p* < 0.001: there was a larger mask advantage for neutral faces than for smiling faces. The one remaining significant interaction was with masks, gender and expression, *F*(1, 16,552) = 7.747, *p* < 0.001. This indicates that when the effect of race is removed, in most cases (all males and neutral females) there is a mask advantage for attractiveness, but for smiling females there is a mask detriment for attractive.

The results show that while there is an overall positive effect of masks on attractiveness, this effect is moderated by the expression, race and gender of the face. The most positive mask effect was for White male faces with a neutral expression—which happens to be the set of stimuli used by Hies and Lewis (2022) to show the positive effect of masks originally. The most negative mask effect was for smiling Black female faces. The conclusion drawn from this analysis therefore is that race, expression and gender all affect the size of the mask effect. There is another way, however, to look at the data.

### Secondary analysis

**Fig. 3:**
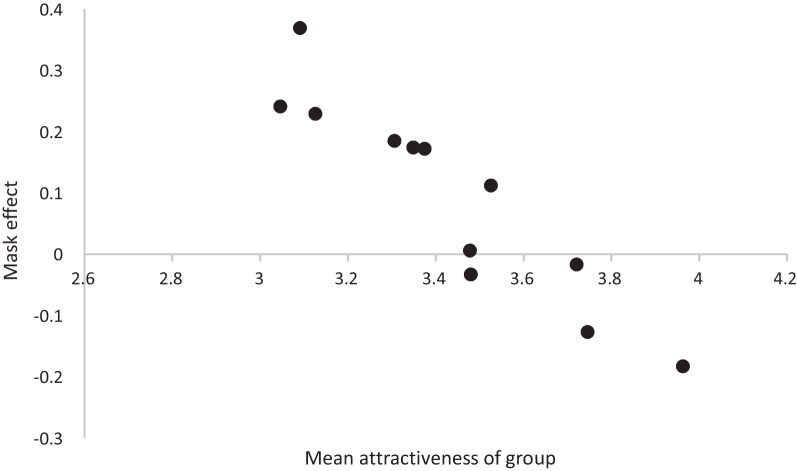
Size of the face mask effect (positive values show an increase in attractiveness with the masks on compared to no masks) for each of the expression-by-race-by-gender categories plotted against the average rated attractiveness of those categories. A clear negative correlation can be observed. “Mask effect” is used as shorthand for effect that mask has on attractiveness such that a positive mask effect means that the mask makes the face more attractive whereas a negative mask effect mean a mask makes the face look less attractive

The largest negative mask effect is for smiling Black female faces, but these also happened to be rated the most attractive faces when unmasked. The largest positive mask effects were for White male faces and these were also rated the lowest for attractiveness when not masked. Indeed, the size of the mask effect strongly correlates, *r*(12) = − 0.929, *p* < 0.001, with the mean attractiveness across the 12 sets of faces (gender-by-race-by-expression) as illustrated in Fig. 3.

This secondary, and admittedly post hoc, analysis suggests that the race, gender and expression effects observed in the primary analysis could, in fact, be merely attractiveness effects. So, for example, smiling faces have a less positive mask effect only because they are more attractive when unmasked than neutral faces. So the important question that can be asked is whether there remain effects of race, expression or gender once the effects of unmasked attractiveness are partialled out.

**Fig. 4:**
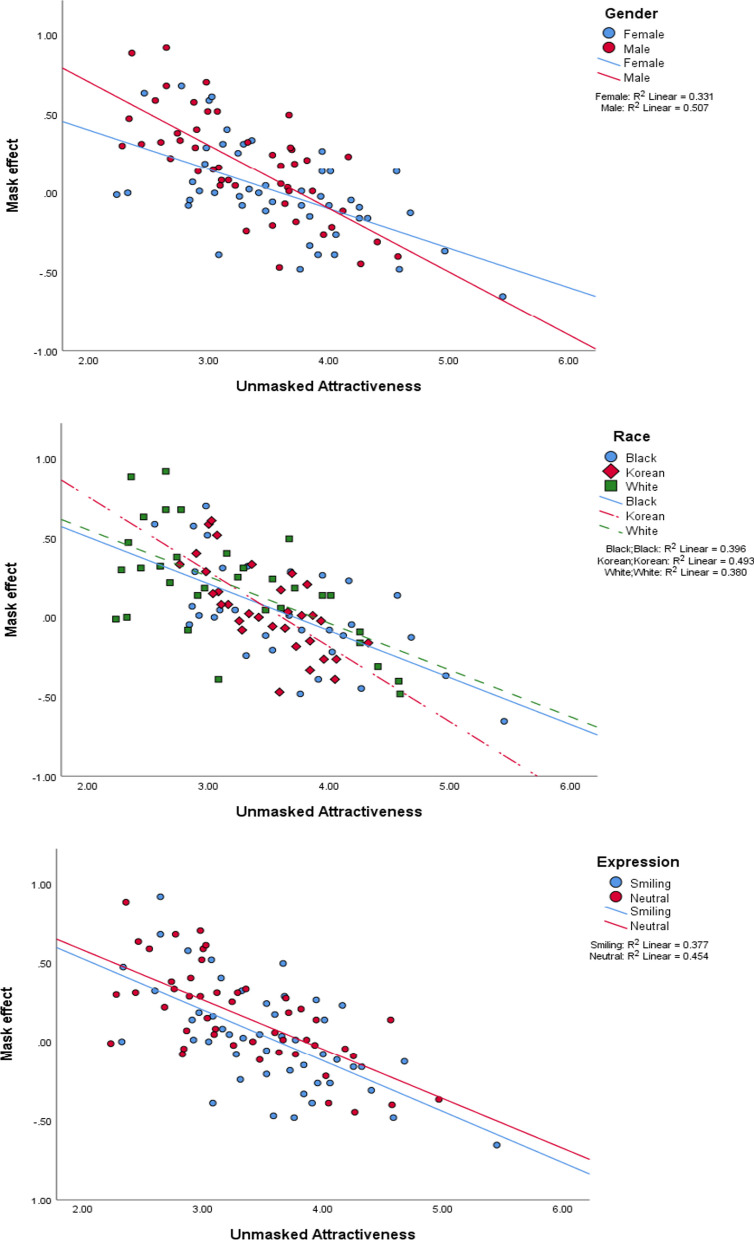
Plots of the size of the mask effect as predicted by unmasked attractiveness split by gender, race or expression. The points represent the 96 masked and unmasked pairs of faces. So, the three panels show the same data but split according to either gender (top panel), race (middle panel) or expression (bottom panel). The top panel illustrates that the correlation between the mask effect and unmasked attractiveness is stronger for male faces than female faces and the middle panel show that the correlation is slightly stronger (but not significantly) for Korean faces than other races. The bottom panel shows that the mask effect is consistently larger for neutral faces than for smiling faces regardless of the base attractiveness of the faces. “Mask effect” is used as shorthand for effect that mask has on attractiveness such that a positive mask effect means that the mask makes the face more attractive whereas a negative mask effect mean a mask makes the face look less attractive

An alterative analysis was carried out on the full dataset, but this time, unmasked attractiveness was included as a predictor. This term was calculated by averaging the attractiveness scores for all participants for each face when it was unmasked. The attractiveness ratings were averaged over participants to provide a more robust measure of unmasked attractiveness than using the individual ratings. A new LMM analysis was conducted including the new predictors derived from unmasked attractiveness. The additional predictors were unmasked attractiveness, the interaction of this with the presence or absence of masks, and the interactions of these first two with each of gender, expression and race. This model produced a better fit to the overall data with and improved AIC value of 53,617. Unmasked attractiveness was a significant predictor of attractiveness, *F*(1, 54.29) = 770.4, *p* < 0.001, as would be expected. The interaction of unmasked attractiveness and mask presence was significant, *F*(1, 16,544.1) = 77.39, *p* < 0.001, indicating that the mask advantage was larger for less attractive unmasked faces than for more attractive faces. Including unmasked attractiveness as a factor in this way affected the previously significant interactions. The mask-by-expression interaction remained significant, *F*(1, 16,544.1) = 3.916, *p* = 0.048, as did the gender-by-mask interaction, *F*(1, 16,544.1) = 11.04, *p* < 0.001. These findings indicate that the findings that male faces and neutral faces show larger positive mask effects are not being carried entirely by the fact that male and neutral faces are less attractive. The three-way gender-by-expression-by-mask interaction remained significant, *F*(1, 16,544.1) = 6.052, *p* = 0.014. However, the race-by-mask interaction was no longer significant in the presence of the unmasked attractiveness effects, *F*(2, 16,544.1) = 1.021, *p* = 0.360. This suggests that the unmasked attractiveness can explain the effect of race on the mask effect indicating that the reason why a race effect is observed is because the faces of different races differ in their attractiveness. Finally, the three-way mask-by-gender-by-unmasked attractiveness was also significant, *F*(1, 16,544.1) = 8.583, *p* = 0.003, showing that the effect of unmasked attractiveness on the mask advantage was greater for male faces than for female faces (see Fig. 4).

The conclusion from this analysis is that the mask advantage is larger for men than for women, and it is reduced in size when a person is smiling. It initially appeared that race influenced the size of the mask advantage but if the effect of attractiveness of individual faces is removed from the analysis, then this race effect disappears. So, although one race may appear to have a larger mask advantage than another, this is merely a feature of the faces selected from that race being less attractive and therefore showing a larger mask effect. It would be expected that if the faces were matched for attractiveness across races, then there would not be an effect of race on the size of the mask effect.

### Re-examining previous findings

The LMM method of analysis offers the opportunity to consider the effect of attractiveness at the item level rather than group level. This can be used to re-evaluate previous research into the mask advantage for attractiveness. Three studies were re-assessed: Kamatani et al. (2023), Dudarev et al. (2022), and Hies and Lewis (2022).

A race effect on the size of the mask advantage had been found previously by Kamatani et al. (2023). They used faces from a variety of races and also selected individuals who were high, medium or low attractiveness within those groups. Even with this attractiveness manipulation, it can be seen that for example, Japanese faces are the most attractive and show the lowest mask advantage, whereas non-Japanese Asian faces were seen as the least attractive and showed the highest mask advantage. This suggests that it is possible that the effect that is reported as a race effect is in fact related to the relative attractiveness of the different faces used. This possibility can be explored by re-analysing their data while using unmasked attractiveness as a predictor. This way, it can be explored whether the race effect on the mask advantage persists if unmasked attractiveness is partialled out.

The data from the Kamatani et al. (2023) were combined into a single data file in narrow format with each row representing a single response. The responses were coded according to participant number, face, and race of the face, whether the image was wearing a mask or not. A further variable was generated as the average rated attractiveness of that face when viewed without a mask. This variable was significantly predicted by race such that some races were more attractive than others, *F*(4,2) = 139.01, *p* = 0.007, indicating that attractiveness was not matched across groups of faces These data were subjected to a LMM analysis with random factors of participant and face. The factorial fixed effects were race of the face, presence of a mask and the average attractiveness of the face when unmasked.

Overall, the effect of masks was significant,* F*(1, 9470.1) = 93.96, *p* < 0.001, and unsurprisingly, the effect of attractiveness when unmasked was significant, *F*(1, 188.4) = 1492.4, *p* < 0.001. The interaction between the effect of masks and unmasked attractiveness was significant, *F*(1, 9479.2) = 25.165, *p* < 0.001), indicating that the size of the improvement observed for masks was smaller for those people who were rated as more attractive without a mask. The interaction between masks and race was not significant in the presence of the interaction between attractiveness and masks, *F*(3, 9442.9) = 2.427, *p* = 0.064. These findings are consistent with the idea that the differences observed in size of the mask effect between races is being driven by the differences in the attractiveness of individuals chosen to represent that race. The re-analysis of the data is available here https://osf.io/qywxt/.

Dudarev et al. (2022) also suggested that race affected the size of the mask effect in spite of taking into account base attractiveness levels. Their data set can also be analysed using actual unmasked attractiveness levels but only at the category level rather than the item level because the raw data were not provided. The six categories of faces were Japanese or White crossed with high, medium or low base attractiveness. For each of these categories, scores were obtained for unmasked attractiveness and the size of the mask effect. The size of the mask effect was regressed against unmasked attractiveness and a dummy variable representing race. The mask effect was significantly predicted by unmasked attractiveness, *t*(3) = − 5.312, *p* = 0.013, while the effect of race was no longer significant once unmasked attractiveness was removed, *t*(3) = 2.922, *p* = 0.061. Including the interaction did not change the pattern of significance. This demonstrates that at least at the category level, race does not significantly influence the size of the mask effect beyond the effect of unmasked attractiveness. The re-analysis of the are available here https://osf.io/qywxt/.

The current data and these two re-analyses show that attractiveness is a main driver of the size of the mask advantage. However, Hies and Lewis (2022) failed to find a mask-by-base-attractiveness interaction on attractiveness suggesting that attractiveness did not have a large effect on the mask advantage. Hies and Lewis (2022) used the base attractiveness ratings from Chicago Face Database rather than the unmasked attractiveness, so they were ratings taken from people not taking part in the experiment. A re-analysis of their data was performed using unmasked attractiveness of the faces as a fixed factor. This re-analysis found a significant interaction between mask wearing and unmasked attractiveness on rated attractiveness, *F*(1, 2878.2) = 9.555,* p* = 0.002. Hence, a previous study that found no effect of attractiveness on the mask advantage, does so this effect if re-analysed with attractiveness taken from the ratings taken within the study.

One limitation of this type of research is that the images created are always two-dimensional. Whether the same findings persist in three-dimensional renders of faces, and face coverings provide an interesting future research question.

## Conclusion

The current study explored the presence of the mask advantage on attractiveness for faces that varied in expression, gender and race. Neutral faces tended to have a larger mask advantage than smiling faces. This effect can be explaining by the fact that smiling increases attractiveness and so by obscuring most of this expression, the advantage of smiling is mostly lost in masked faces. The mask advantage is greater for male faces than for female faces. This is harder to explain but one could speculate that it may be that the eyes are more important in the evaluation of female faces whereas the jaw line may be more import for male faces. So, the gender difference in the mask advantage can be explained by them obscuring features that are more important in the evaluation of one gender over the other.

Race has previously been reported to be a factor determining the size of the mask effect even when base attractiveness is used to select the face used. Here, race was also found to change the size of the mask effect; however, re-analysis of the data with the additional factor of unmasked attractiveness removed any effects of race suggesting that the observed race effects only occur because of differences of attractiveness of the selected items. This level of analysis was only possible using LMM methods, as more traditional ANOVAs pool across stimuli groups. Re-analysis of previously published studies also found that race effects did not survive the addition of unmasked attractiveness as a predictor. The conclusion is, therefore, that any race effect on the size of the mask advantage is a result of the unmasked attractiveness of the faces with the mask advantage being larger for less attractive faces. This finding brings order to the data on the mask advantage and can explain why some studies found a negative effect and most have found positive effects and also why race appeared to affect the size of mask advantage. It remains the case that there is an effect of gender on the mask effect that is beyond that of unmasked attractiveness and gender even interacts with the unmasked attractiveness effect on the effect of masks on attractiveness. Exactly why these gender effects and interactions persist remains an open question.

## Data Availability

The data, analysis and re-analysis of published data are available here https://osf.io/qywxt/.

## Footnotes

1 The racial terms Black, White and Korean are taken from the databases employed. We acknowledge that these labels conflate nationality with inheritable facial properties. The labels are useful, however, to distinguish how the different faces would likely be categorised.
